# Evaluating the role of *MLH3* p.Ser1188Ter variant in inherited breast cancer predisposition

**DOI:** 10.1038/s41436-019-0694-8

**Published:** 2019-11-05

**Authors:** Susanna Koivuluoma, Robert Winqvist, Riikka Keski-Filppula, Outi Kuismin, Jukka Moilanen, Katri Pylkäs

**Affiliations:** 1 Laboratory of Cancer Genetics and Tumor Biology, Cancer and Translational Medicine Research Unit and Biocenter Oulu, Northern Finland Laboratory Centre Nordlab Oulu, University of Oulu, Oulu, Finland; 2 Department of Clinical Genetics, Oulu University Hospital, Medical Research Center Oulu and PEDEGO Research Unit, University of Oulu, Oulu, Finland

Germline loss-of-function variants in mismatch repair (MMR) genes elevate risk for specific cancer types, especially colorectal, endometrial, and gastric carcinomas.^1–3^ Whether MMR gene variants confer an increased risk for breast cancer has been under debate with studies providing evidence for and against this.^4–6^ Recently, Olkinuora et al.^7^ described a germline nonsense variant in DNA mismatch repair gene *MLH3* (MutL Homolog 3), p.Ser1188Ter, which in biallelic form underlies a novel polyposis predisposition syndrome with susceptibility to classical or attenuated adenomatous polyposis and possibly also extracolonic tumors, including breast cancer. In their study, *MLH3* p.Ser1188Ter was identified in four Finnish polyposis families, of which three also exhibited breast cancer. In one family, both cases with breast cancer were homozygous for *MLH3* p.Ser1188Ter, whereas in the other two families the breast cancer cases (in different generations than the identified homozygous index case) were not available for testing.^7^

According to public databases *MLH3* p.Ser1188Ter variant (rs193219754) is enriched in the Finnish population (Finnish minor allele frequency [MAF] 0.0026 versus global MAF 0.00026) with a carrier frequency of 7/1239 (0.6%) in Northern Ostrobothnia (gnomAD, https://gnomad.broadinstitute.org/; SISu, http://www.sisuproject.fi/). Although an otherwise extremely rare allele globally, this enrichment provides a unique setting for testing of its association with breast cancer susceptibility. This is particularly important for genetic counseling, as the use of large gene panels containing a variety of hereditary cancer genes, even when patients lack classic clinical features associated with some of the genes, is becoming routine in the clinics. Here, we have tested the prevalence of the *MLH3* p.Ser1188Ter variant in breast cancer patient cohorts collected from Northern Ostrobothnia (Oulu University Hospital), defined as hereditary cohort (*n* = 225) and unselected breast cancer cohort (*n* = 1083). High-resolution melt analysis (CFX96, Bio-Rad, Hercules, CA, USA) and Sanger sequencing (ABI3130xl, Applied Biosystem, Foster City, CA, USA) were used for genotyping. The hereditary cohort included affected index breast cancer cases from (1) 137 families with three or more breast and/or ovarian cancers in first- or second-degree relatives; (2) 31 families with two cases of breast, or breast and ovarian cancer in first- or second-degree relatives, of whom at least one had early disease onset (<35 years), bilateral breast cancer, or multiple primary tumors including breast or ovarian cancer in the same individual; (3) 46 families with two cases of breast cancer in first- or second-degree relatives; in addition to (4) 11 breast cancer cases diagnosed with breast cancer at or below the age of 40 (median 38, range 25–40 years). The unselected breast cancer cohort consisted of 1083 consecutive breast cancer cases diagnosed at the Oulu University Hospital during the years 2000–2016 (with a mean age at diagnosis 58 years) and were unselected for the family history of cancer and age at disease onset. This study included informed consent from all participating individuals, and it was approved by the Ethical Board of the Northern Ostrobothnia Health Care District.

Only one index case from the hereditary cohort was identified as a carrier (1/225, 0.44%, *P* = 1.0, odds ratio [OR] = 0.79, 95% confidence interval [CI] 0.1–6.4, Supplemental Table 1). She was diagnosed with breast cancer at the age of 43 years. There were five additional breast cancer cases in the family, but only one of them (breast cancer 54 years, melanoma 40 years) was identified to carry *MLH3* p.Ser1188Ter (Supplemental Table 2, Fam#1). As three healthy female family members were also identified as *MLH3* p.Ser1188Ter carriers, there was no evidence for *MLH3* p.Ser1188Ter segregation with the disease within this family. In the unselected cohort, six *MLH3* p.Ser1188Ter carriers were identified (6/1083, 0.55%, *P* = 1.0, OR = 0.98, 95% CI 0.3–2.9, Supplemental Table 1) with mean age at disease onset 63 years (minimum 50 years, maximum 85 years). Of these, three had a family history of other than breast cancer (Supplemental Table 2, Unsel#1–6). The frequency of *MLH3* p.Ser1188Ter in the studied breast cancer cohorts (7/1308, 0.54%, P=1.0, OR = 0.95, 95% CI 0.3–2.7, Supplemental Table 1) did not differ from the population frequency (7/1239, 0.56%) in this geographical region, arguing against association with breast cancer risk. No *MLH3* p.Ser1188Ter homozygotes were observed. Altogether these results indicate that based on the performed case–control comparison *MLH3* p.Ser1188Ter is unlikely to be a significant contributor to breast cancer risk, at least when heterozygous.

## Supplementary information

Supplementary Table 1

Supplementary Table 2
