# Supplementary material for: Evaluating the role of MLH3 p.Ser1188Ter variant in inherited breast cancer predisposition
Source: Genet Med. 2019 Nov 5;22(3):663–4. doi: 10.1038/s41436-019-0694-8 (PMC7056660; doi:10.1038/s41436-019-0694-8)
Supplement: Supplementary file 1 — Supplementary Table 1 [file 41436_2019_694_MOESM1_ESM.docx]

**Supplemental Table 1. Frequency of *MLH3* p.Ser1188Ter variant in the studied breast cancer cohorts and controls**

| **Cohort** | **N** | **WT** | **%** | **Mut** | **%** | **OR** | **95% CI** | **P** ^b^ |
| --- | --- | --- | --- | --- | --- | --- | --- | --- |
| Hereditary | 225 | 224 | 99.56 | 1 | 0.44 | 0.79 | 0.1-6.4 | 1 |
| Unselected | 1083 | 1077 | 99.45 | 6 | 0.55 | 0.98 | 0.3-2.9 | 1 |
| All BC | 1308 | 1301 | 99.46 | 7 | 0.54 | 0.95 | 0.3-2.7 | 1 |
| Controls (SISu)^a^ | 1239 | 1232 | 99.44 | 7 | 0.56 |  |  |  |

BC: breast cancer, CI: confidence interval, Mut: mutation, OR: odds ratio, WT: wildtype

^a^ http://www.sisuproject.fi; rs193219754

^b^ Fisher’s exact test
