# Supplementary material for: Evaluating the role of MLH3 p.Ser1188Ter variant in inherited breast cancer predisposition
Source: Genet Med. 2019 Nov 5;22(3):663–4. doi: 10.1038/s41436-019-0694-8 (PMC7056660; doi:10.1038/s41436-019-0694-8)
Supplement: Supplementary file 2 — Supplementary Table 2 [file 41436_2019_694_MOESM2_ESM.docx]

**Supplemental Table 2. Family history of the identified *MLH3* p.Ser1188Ter carriers**

| Index ID -Cancers/tumors  (age at diagnosis) | Breast/ovarian cancer(s) in 1^st^ and 2^nd^  degree relatives | Other cancers (age at diagnosis) in 1^st^ and 2^nd^ degree relatives |
| --- | --- | --- |
| Fam#1 - Br (43) | Br (54)+Mel (40)[+], Br (53) [-], Br (49)[-], Br (u)[-], Bil Br (55)+Lymph (u)[-] | - |
| Unsel#1 - Br (67) | - | Uterine (u), prostate (u), brain (u), stomach (30) |
| Unsel#2 - Br (62) | - | Kidney (62), rectal (45), kidney (60) |
| Unsel#3 - Br (51), Bil Br (65) | - | Brain (u), brain (39) |
| Unsel#4 - Br (85) | - | - |
| Unsel#5 - Br (65) | - | - |
| Unsel#6 - Br (50) | - | - |

- : none reported, Br: breast cancer, Bil Br: bilateral breast cancer, Lymp: lymphoma, Mel: melanoma, u: age at diagnosis unknown

[+] tested positive, or tested [-] negative for *MLH3* p.Ser1188Ter
